# Oral Barrier Immunometabolism in Chronic Low-Grade Inflammation: Molecular Mechanisms and Systemic Implications

**DOI:** 10.3390/ijms27125356

**Published:** 2026-06-13

**Authors:** Aferdita Ademi, Skender Topi, Mitilda Gugu, Alessia Ciafarone, Maria Grazia Cifone, Davide Pietropaoli, Serena Altamura

**Affiliations:** 1 Faculty of Medical Technical Sciences, University of Elbasan “Aleksandër Xhuvani”, 3001 Elbasan, Albania; ademidita@gmail.com (A.A.); skender.topi@uniel.edu.al (S.T.); mitilda.gugu@uniel.edu.al (M.G.); 2 Department of Physical & Chemical Sciences, University of L’Aquila, 67100 L’Aquila, Italy; alessia.ciafarone@univaq.it; 3 Department of Health & Environment Sciences, University of L’Aquila, 67100 L’Aquila, Italy; mariagrazia.cifone@univaq.it (M.G.C.); davide.pietropaoli@univaq.it (D.P.)

**Keywords:** oral mucosa, immunometabolism, oral microbiota, NLRP3 inflammasome, mtROS, barrier tissues, chronic inflammation, oral–systemic axis, inflammaging

## Abstract

Chronic low-grade inflammation is a hallmark of aging and a major driver of metabolic and degenerative diseases. While systemic immune dysfunction has been widely investigated, the contribution of barrier tissues to persistent inflammatory signaling remains incompletely defined. The oral mucosa represents a uniquely exposed barrier, continuously challenged by microbial, mechanical, and metabolic stressors and characterized by a specialized immune architecture. Here, we synthesize current evidence supporting the oral barrier as an active immunometabolic interface linking local immune activation to systemic inflammatory tone. Spatially organized epithelial, neutrophil, and antigen-presenting cell (APC) compartments coordinate immune responses tightly coupled to metabolic reprogramming, including hypoxia-inducible factor-1α (HIF-1α)-dependent glycolysis and mitochondrial reactive oxygen species (mtROS) production. In parallel, the oral microbiota provides ligands and metabolites such as lipopolysaccharide (LPS), short-chain fatty acids (SCFAs), and succinate, which activate pattern-recognition receptors (PRRs), including toll-like receptors (TLRs) and the NOD-like receptor pyrin domain-containing 3 (NLRP3) inflammasome, thereby sustaining nuclear factor kappa-light-chain-enhancer of activated B cell (NF-κB)-mediated inflammatory signaling. Barrier disruption and dysbiosis promote microbial translocation and persistent innate immune activation, while saliva and gingival crevicular fluid facilitate systemic dissemination of inflammatory mediators. Overall, sustained immunometabolic engagement at the oral barrier emerges as a key driver of chronic low-grade systemic inflammation and a potential therapeutic target in inflammaging.

## 1. Introduction

Chronic inflammation is a central pathogenic mechanism underlying a wide spectrum of age-associated diseases, including metabolic disorders, cardiovascular disease, neurodegeneration, and cancer. Unlike acute inflammation, which is tightly regulated and self-resolving, chronic low-grade inflammation persists over time due to sustained immune activation, metabolic stress, and impaired resolution pathways. Increasing evidence indicates that this form of inflammation arises not only from systemic immune dysregulation but also from localized inflammatory signals originating at barrier tissues that progressively shape organism-wide immune tone [1,2].

Barrier surfaces such as the gastrointestinal tract, skin, lungs, and oral mucosa are continuously exposed to microbial, mechanical, and environmental stressors [3]. To cope with this constant challenge, these tissues have evolved highly specialized immune architectures that integrate innate sensing, immune regulation, and metabolic adaptability [4]. However, chronic or repetitive stimulation at barrier sites can overwhelm these adaptive mechanisms, leading to persistent activation of innate immune pathways and sustained production of inflammatory mediators [5]. In this context, barrier tissues are increasingly recognized not merely as passive targets of inflammation but as active sources and amplifiers of chronic inflammatory signaling.

Among barrier sites, the oral mucosa represents a unique immunological environment [6,7]. It is subjected to exceptionally high microbial exposure, frequent mechanical disruption, and continuous metabolic fluctuation, while simultaneously maintaining remarkable resistance to overt tissue damage. Recent advances in spatial biology and mucosal immunology have revealed that the oral barrier is organized into distinct immunological compartments. These include epithelial layers specialized for immune sensing, neutrophil-rich interfaces that provide front-line defense, and deeper aggregates of antigen-presenting cells (APCs) and lymphocytes coordinating adaptive responses. This spatial organization supports persistent immune surveillance while also creating conditions in which inflammatory signaling can become chronically engaged [8].

In parallel, the oral microbiota constitutes one of the most diverse and metabolically active microbial ecosystems in the human body [9]. Oral microbial communities produce a wide range of bioactive metabolites and inflammatory ligands capable of engaging pattern-recognition receptors (PRRs), activating nuclear factor kappa-light-chain-enhancer of activated B cells (NF-κB)-dependent pathways, and modulating host cellular metabolism [10]. Dysbiosis, epithelial stress, and impaired barrier integrity enhance microbial translocation and amplify innate immune activation, thereby favoring the persistence of low-grade inflammation [2,9,11].

At the molecular level, barrier-associated inflammatory responses are tightly regulated by interconnected metabolic and signaling pathways. Persistent activation of PRRs, including toll-like receptors (TLRs) and nucleotide-binding oligomerization domain (NOD)-like receptors (NLRs), intersects with key metabolic regulators such as hypoxia-inducible factor-1α (HIF-1α) and mechanistic target of rapamycin (mTOR), driving shifts toward glycolysis and sustained cytokine production [12,13]. Mitochondrial dysfunction and reactive oxygen species (ROS) generation further contribute to the activation of the NLR family pyrin domain-containing 3 (NLRP3) inflammasome, linking metabolic stress to chronic interleukin (IL)-1β-mediated inflammatory signaling. Microbiota-derived metabolites, including short-chain fatty acids (SCFAs) and succinate, also modulate these pathways through specific receptors, reinforcing the coupling between immune activation and cellular metabolism at barrier tissues. Together, these processes suggest that sustained immunometabolic interactions at the oral barrier may contribute to chronic inflammation beyond the local tissue environment.

Saliva and gingival crevicular fluid further integrate the oral barrier into systemic inflammatory networks. These biofluids contain cytokines, immune cells, microbial components, and metabolites that reflect ongoing mucosal immune activity and facilitate the dissemination of oral-derived inflammatory signals [14,15]. Through these mechanisms, localized inflammatory processes at the oral mucosa may influence systemic immune activation and contribute to the persistence of chronic inflammation [16]. In this review, we examine inflammatory and immunometabolic mechanisms operating at the oral barrier, focusing on epithelial metabolic reprogramming, microbiota-driven innate immune signaling, and barrier-derived pathways that sustain chronic low-grade inflammation. By framing the oral mucosa as a model barrier tissue, we highlight general principles through which barrier immunometabolism contributes to systemic inflammation during aging. Existing models such as the oral–systemic axis have largely focused on microbial dissemination and circulating inflammatory mediators. Building on these frameworks, the present work introduces an additional layer of complexity by emphasizing local immunometabolic processes within the oral mucosa. In this view, the oral barrier is not only a source of systemic signals but also an active site of integrated metabolic and immune regulation that may contribute to persistent inflammation. Accordingly, we propose a unifying framework in which the oral mucosa functions as a spatially organized immunometabolic interface, linking local barrier activity to systemic inflammatory regulation and contributing to the maintenance of chronic low-grade inflammation.

## 2. Immune Architecture of the Oral Mucosa

The oral mucosa is a highly specialized barrier tissue that must maintain immune equilibrium while continuously facing exposure to microbial communities, mechanical stress, and environmental perturbations [17]. To meet this challenge, the oral barrier has evolved a complex and spatially organized system that integrates innate sensing, immune regulation, and rapid inflammatory responsiveness.

The oral mucosa exhibits a compartmentalized immune architecture, in which distinct immunological zones support persistent surveillance while limiting excessive tissue damage.

### 2.1. Immune Zonation and Spatial Organization

**Figure 1:**
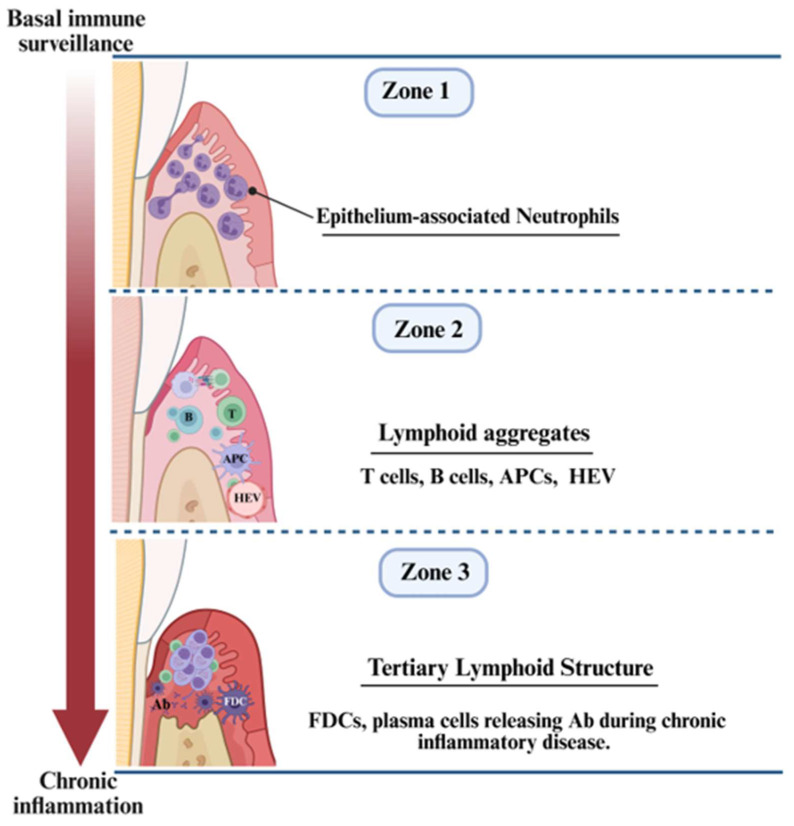
Immune zonation of the human oral mucosa. The oral mucosa exhibits a progressive spatial organization of immune compartments, reflecting increasing levels of immune activation and inflammatory complexity. Zone 1 represents the superficial epithelial compartment enriched in epithelium-associated neutrophils that provide continuous immune surveillance and rapid innate defense. Zone 2 comprises organized lymphoid aggregates containing T cells, B cells, and APCs, supported by specialized vasculature including HEVs, which facilitate immune cell recruitment and coordination of adaptive immune responses. Zone 3 depicts the development of tertiary lymphoid structures under conditions of chronic inflammation, characterized by FDCs and Ab-producing plasma cells. Together, these spatially defined immune zones constitute a functional framework that sustains persistent immune engagement while shaping inflammatory dynamics at the oral barrier. Created in BioRender. Palumbo, P. (2026) https://BioRender.com/ks46uk1 (accessed on 7 May 2026).

Recent advances in spatial biology and high-resolution imaging have revealed that the oral mucosa is organized into a dynamically stratified immune landscape, in which distinct yet interconnected compartments coordinate host responses across physiological and inflammatory conditions [6]. This spatial arrangement reflects a dynamic gradient of immune activation extending from the epithelial surface to deeper mucosal layers (Figure 1).

At the host–microbiota interface, the superficial epithelial layer forms the primary frontline of immune activity. This compartment is enriched in PRRs and antimicrobial effectors and is closely associated with epithelium-associated neutrophils, which provide continuous microbial surveillance and rapid innate immune responses, while preserving tissue integrity and preventing excessive inflammatory damage [18].

Proceeding deeper into the mucosa, the immune landscape becomes progressively more structured. Localized aggregates of antigen-presenting cells (APCs) and lymphocytes form organized niches that coordinate adaptive immune responses [19]. These aggregates are supported by specialized stromal and vascular elements, including high endothelial venules (HEVs), which facilitate immune cell recruitment and enhance local immune activation [20].

Under conditions of sustained or chronic stimulation, these organized immune niches progressively evolve toward tertiary lymphoid structures [21,22]. This transition reflects increasing immune activation and chronicity and is characterized by the presence of follicular dendritic cells (FDCs) and plasma cells capable of local antibody (Ab) production [22]. In this context, spatial organization not only defines compartmentalization but also captures the dynamic maturation of immune responses across the epithelial–submucosal axis.

Importantly, this zonation is preserved across both physiological and pathological conditions, indicating that it represents a fundamental organizational principle of oral mucosal immunity. Stromal cells play a central role in maintaining this architecture by providing location-specific transcriptional and metabolic cues that shape epithelial and immune cell behavior and sustain compartment-specific immune functions [23].

Collectively, this spatially organized immune system enables the oral mucosa to sustain continuous immune surveillance while limiting excessive inflammatory escalation. At the same time, progressive transitions across these compartments provide a structural framework through which persistent stimulation can drive chronic immune activation, ultimately contributing to sustained low-grade inflammatory signaling.

### 2.2. Resident Immune Cell Populations and Inflammatory Regulations

The oral mucosa hosts a dense and diverse population of resident immune cells that collectively regulate inflammatory tone at the barrier surface [19]. Neutrophils are continuously recruited to the gingival sulcus and epithelial interface, where they form a dominant component of barrier immunity. Unlike neutrophils in other tissues, oral neutrophils function under conditions of constant microbial exposure and are adapted to limit excessive inflammation while maintaining antimicrobial defense [24]. However, chronic recruitment and sustained activation of these cells can contribute to prolonged release of inflammatory mediators and increased oxidative stress [24].

Beneath the epithelial surface, dendritic cells and macrophages act as central integrators of microbial, metabolic, and mechanical signals. These APCs continuously sample the local environment and translate persistent stimulation into inflammatory or regulatory outputs that shape downstream immune responses [25]. Tissue resident lymphocyte populations, including T cells, B cells, and innate lymphoid cells, further modulate inflammatory dynamics through cytokine production, immune regulation, and maintenance of epithelial integrity. The activity of these immune populations is closely linked to local metabolic cues and microbiota-derived metabolites, reinforcing the interplay between inflammation and immunometabolism at the oral barrier.

Through the coordinated activity of these resident immune populations, the oral mucosa maintains a state of heightened inflammatory readiness that supports barrier protection but may increase susceptibility to sustained immune activation when regulatory mechanisms fail.

### 2.3. Saliva as an Immunological and Inflammatory Interface

Saliva represents a dynamic extension of oral mucosal immunity and functions as an active immunological medium rather than a passive fluid. Within the oral barrier environment, it contains epithelial cells, neutrophils, monocytes, dendritic cells, and lymphocyte subsets, together with a wide array of cytokines, chemokines, antimicrobial peptides, and secretory immunoglobulin A (sIgA). Together, these components contribute to microbial containment, immune regulation, and inflammatory signaling at the mucosal surface.

Gingival crevicular fluid, derived from serum exudate and periodontal tissues, further enriches this local milieu with inflammatory mediators, immune cells, and microbial products. Importantly, both saliva and gingival crevicular fluid reflect real-time immune activity at the oral barrier and serve as dynamic readouts of ongoing immunological and metabolic processes within the mucosal environment [15,26].

By acting as mobile immune compartments, these biofluids integrate epithelial and immune responses across the oral barrier, supporting localized coordination of host–microbiota interactions while also providing potential routes for systemic dissemination of inflammatory signals.

## 3. Oral Microbiota and Immune–Metabolic Regulation

The oral cavity hosts one of the most diverse and metabolically active microbial ecosystems in the human body. Rather than acting as a passive microbial reservoir, the oral microbiota establishes continuous interactions with epithelial and immune cells at the barrier surface, which may contribute to the regulation of local and systemic inflammatory responses. A key feature of these interactions is their potential to sustain immunometabolic activation over time, favoring persistent inflammatory signaling rather than transient, self-limiting host defense responses.

### 3.1. Composition and Immunometabolic Activity of the Oral Microbiota

Beyond compositional diversity, the functional impact of the oral microbiota on host physiology lies in its ability to modulate immune and metabolic pathways. Specific taxa, including *Porphyromonas gingivalis*, *Fusobacterium nucleatum*, *Tannerella forsythia*, and *Treponema denticola*, act as pathobionts capable of disrupting epithelial integrity and activating innate immune signaling [2,27,28]. These microorganisms produce virulence factors and bioactive molecules that engage PRRs such as TLRs and NLRs, thereby promoting NF-κB–dependent transcriptional programs. In parallel, microbial signaling may induce metabolic reprogramming in host cells, often characterized by a shift toward glycolysis mediated by HIF-1α stabilization.

Importantly, these effects extend beyond immune activation to include alteration in epithelial barrier function, redox balance, and mitochondrial activity. Through this coordinated influence on immune signaling and cellular metabolism, the oral microbiota may act as a key regulator of barrier immunometabolic homeostasis.

### 3.2. Microbial Metabolites as Drivers of Chronic Inflammatory Signaling

A defining mechanism through which the oral microbiota influences host physiology is the production of metabolites that function as signaling molecules at the host–microbe interface. These include SCFAs, amino acid derivatives, nitrogenous compounds, and redox-active molecules [29]. Under homeostatic conditions, SCFAs such as acetate and butyrate exert regulatory effects by engaging G protein–coupled receptors (e.g., GPR43) and promoting anti-inflammatory pathways, including IL-10 production and maintenance of epithelial barrier integrity. However, dysbiosis alters microbial metabolic output, reducing beneficial metabolites while increasing pro-inflammatory intermediates. Among these, succinate has emerged as a key immunometabolic signal [30,31]. Its accumulation stabilizes HIF-1α, enhances cytokine production, and amplifies innate immune responses through receptor-mediated pathways. Similarly, microbial-derived molecules such as trimethylamine N-oxide (TMAO) and LPS have been linked to NF-κB activation and inflammasome signaling [32,33,34]. However, circulating TMAO levels are determined by a complex multi-organ pathway involving intestinal microbial production of trimethylamine (TMA), hepatic conversion via flavin-containing monooxygenase 3 (FMO3), and renal clearance [35,36]. In this context, the specific contribution of the oral microbiota to systemic TMAO levels remains incompletely defined.

Many of these metabolites engage pathways associated with inflammaging [37,38], including NF-κB activation, increased IL-6 and tumor necrosis factor (TNF) production, and elevated oxidative stress. In addition, several microbial metabolites impair mitochondrial function, leading to increased mtROS production [39], which act as a secondary signal for NLRP3 inflammasome activation. Through these mechanisms, microbial metabolites may link dysbiosis, metabolic stress, and chronic inflammatory signaling. Dysbiosis-driven shifts in microbial metabolic output have been associated with systemic inflammation and degenerative conditions, further supporting the role of microbial metabolites as molecular mediators connecting oral disturbances to organism-wide inflammatory burden [40].

Altogether, microbiota-derived metabolites may act as systemic effectors that reinforce chronic low-grade inflammation and contribute to age-related immunometabolic decline. In this context, they emerge as key amplifiers of inflammatory signaling beyond the oral cavity.

### 3.3. Biofilm Architecture as a Persistent Inflammatory Niche

Oral biofilms are highly organized three-dimensional structures embedded within an extracellular matrix that regulates nutrient diffusion, oxygen gradients, and metabolite exchange [41,42]. This complex architecture promotes microbial persistence and concentrates inflammatory ligands at the epithelial interface. As a result, immune cells interacting with biofilms encounter dense arrays of microbial products and metabolites, leading to localized yet sustained immune activation.

Biofilm organization favors chronicity by maintaining prolonged host-microbe interactions, limiting effective clearance, and promoting repeated innate immune stimulation. Under dysbiotic conditions, this localized inflammatory engagement can extend into systemic circulation, further amplifying inflammatory tone. Accordingly, biofilms function not merely as reservoirs of microbes but as stable inflammatory microenvironments that sustain immunometabolic stress [43].

### 3.4. Oral Microbiota as a Source of Systemic Immune Activation

**Figure 2:**
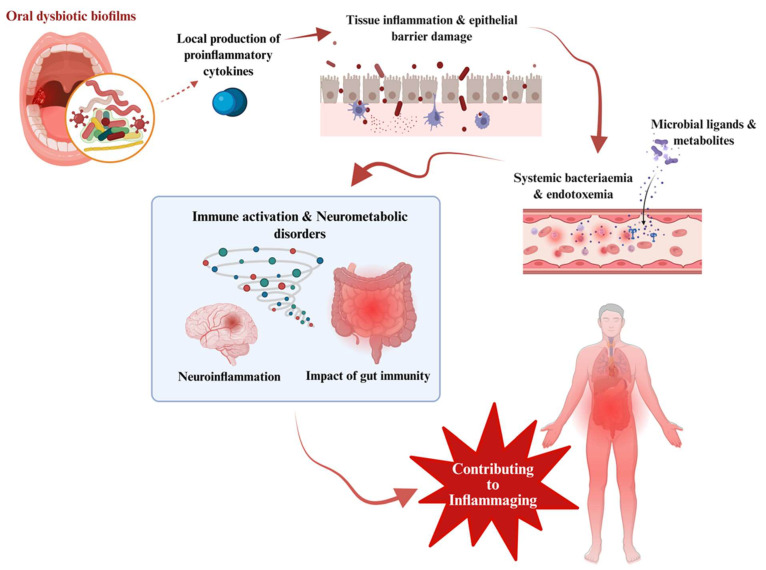
Oral dysbiotic biofilms induce epithelial barrier damage and local inflammation, promoting microbial translocation and systemic immune activation. These events contribute to neuroinflammation, altered gut immunity, and inflammaging. Created in BioRender. Palumbo, P. (2026) https://BioRender.com/ks46uk1 (accessed on 7 May 2026).

The impact of the oral microbiota extends beyond the local tissue environment through mechanisms that link oral inflammation to systemic immune responses. Dysbiotic communities promote the local production of cytokines and inflammatory mediators that can enter the circulation, thereby contributing to organism-wide inflammatory tone [44,45,46,47,48,49,50,51,52] (Figure 2). Compromised epithelial integrity further facilitates the translocation of microbial components, including LPS and other ligands capable of activating circulating immune cells. Even low-level, repeated exposure to these signals is sufficient to sustain chronic activation of innate immune pathways [53,54]. 

In addition, microbial metabolites act as diffusible mediators that influence distal tissues, modulating metabolic and inflammatory processes in organs such as the liver, vasculature, and central nervous system. These processes contribute to interconnected immunometabolic networks, including the oral–gut and oral–systemic axes [55].

Collectively, these mechanisms indicate that the oral microbiota functions not only as a local ecological community but also as a systemic immunometabolic regulator capable of sustaining chronic low-grade inflammation.

## 4. Oral Epithelial Immunometabolism

The oral epithelium represents a dynamic interface that integrates microbial exposure, mechanical stress, and inflammatory signals within a tightly regulated immunometabolic framework. Beyond its structural role, this barrier is metabolically active and continuously adapts its bioenergetic state to sustain antimicrobial defense, tissue repair, and immune signaling. Under physiological conditions, these adaptive responses support barrier resilience. However, persistent stimulation can reprogram epithelial immunometabolism toward chronic inflammatory activation, thereby contributing to the maintenance of low-grade systemic inflammation.

### 4.1. Metabolic Reprogramming Underlying Epithelial Inflammatory Responses

**Figure 3:**
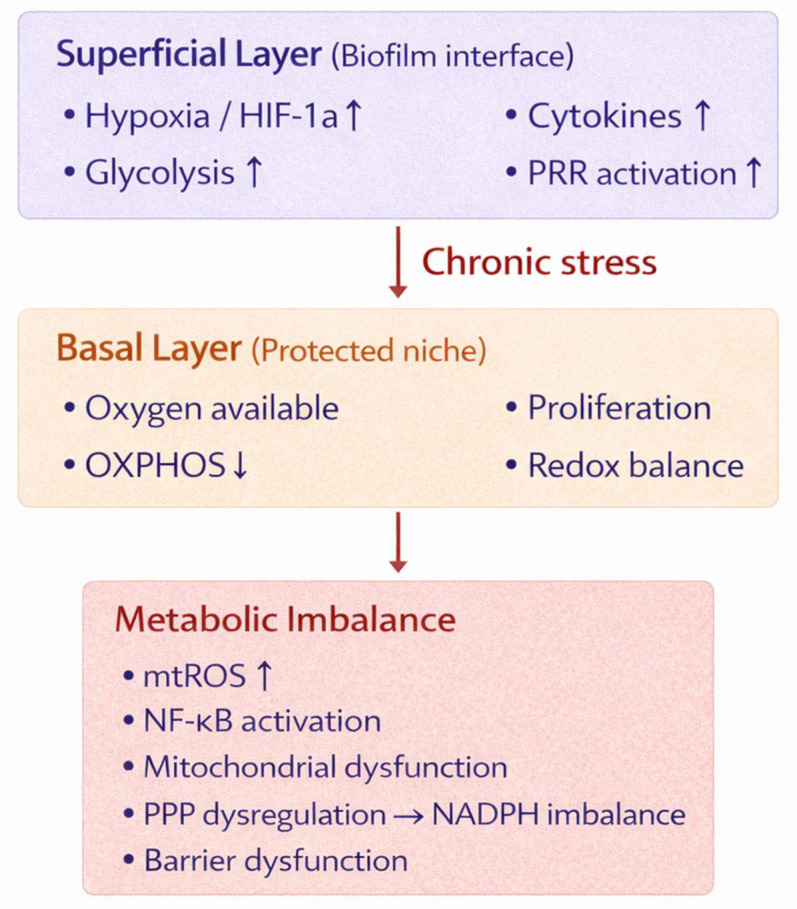
Spatial immunometabolic compartmentalization and dysfunction in oral epithelium. The oral epithelium exhibits a spatially organized metabolic architecture, with superficial layers at the biofilm interface characterized by hypoxia, HIF-1α stabilization, increased glycolysis, and PRR activation induced by microbial ligands. In contrast, basal epithelial layers reside in a protected niche with higher oxygen availability and rely predominantly on oxidative phosphorylation, supporting proliferation and redox homeostasis. Under conditions of chronic stress, this compartmentalization is disrupted, leading to metabolic imbalance characterized by mitochondrial dysfunction, increased mtROS, sustained NF-κB activation, and impaired barrier integrity. Concomitantly, dysregulation of the PPP alters NADPH availability, compromising antioxidant defenses and further contributing to redox imbalance and chronic inflammatory signaling.

Oral epithelial cells exhibit marked metabolic plasticity, enabling rapid transitions between oxidative phosphorylation and glycolysis in response to microbial and inflammatory cues. Activation by microbial ligands and cytokines promotes HIF-1α stabilization, inducing a glycolytic program characterized by increased expression of glucose transporters, such as glucose transporter type 1 (GLUT1), and key glycolytic enzymes. This metabolic shift supports the adenosine triphosphate (ATP) production required for cytokine secretion and antimicrobial peptide synthesis [12,13,56]. This metabolic reprogramming is spatially regulated across the epithelial layers. Superficial cells exposed to oral biofilms adopt a predominantly glycolytic phenotype, whereas deeper layers retain higher mitochondrial activity and oxidative metabolism. Such compartmentalization enables rapid frontline responses while preserving proliferative capacity and redox balance in basal compartments. The preference for glycolysis in superficial epithelial layers reflects both extrinsic and intrinsic constraints. At the epithelial–biofilm interface, oxygen availability is limited due to microbial consumption and diffusion barriers within the biofilm matrix, creating a relatively hypoxic microenvironment that stabilizes HIF 1α and promotes glycolytic metabolism [57,58]. Rapid ATP generation through glycolysis supports antimicrobial responses and cytokine secretion [59,60]. It also contributes to barrier integrity under continuous microbial challenge [61]. In contrast, deeper epithelial layers are less exposed to hypoxia and inflammatory stimuli and maintain higher mitochondrial activity, supporting oxidative metabolism required for proliferation, differentiation, and long-term tissue homeostasis [60,62]. This spatial organization enables the epithelium to balance immediate defense with tissue renewal and redox control [60]. Disruption of this compartmentalization leads to epithelial dysfunction, with excessive glycolysis, impaired mitochondrial activity, and loss of redox balance. This imbalance promotes accumulation of mtROS, sustained NF κB activation, and chronic inflammasome signaling, ultimately leading to impaired barrier function and increased permeability. Consequently, the epithelium shifts from a regulated adaptive state to a persistent pro-inflammatory condition (Figure 3).

However, sustained HIF-1α activation and prolonged reliance on glycolysis can impose metabolic stress. Chronic alterations in the nicotinamide adenine dinucleotide (NAD^+^)/nicotinamide adenine dinucleotide reduced form (NADH) balance, together with impaired mitochondrial function, lead to increased production of mtROS. Although mtROS act as important signaling mediators, their persistent elevation can promote epithelial dysfunction and inflammatory stress. Through these mechanisms, epithelial metabolic reprogramming shifts from an adaptive to a maladaptive state, ultimately favoring persistent inflammatory signaling [39].

### 4.2. Pattern-Recognition Receptor Signaling and Metabolic Control of Inflammation

PRRs, including TLRs and NLRs, are constitutively expressed by oral epithelial cells and act as primary integrators of microbial and metabolic signals [63]. Engagement of these receptors activates NF-κB and mitogen-activated protein kinase (MAPK) signaling pathways, which are tightly coupled to metabolic reprogramming [64]. TLR-mediated signaling promotes a shift toward glycolysis, thereby supporting inflammatory gene transcription and epithelial effector functions. In parallel, mTOR signaling coordinates nutrient sensing, protein synthesis, and epithelial proliferation, linking metabolic availability to immune responsiveness. Together, these pathways establish a metabolically “primed” epithelial state that enables rapid responses to microbial challenge.

A critical molecular node connecting metabolic stress to inflammation is represented by the NLRP3 inflammasome [65,66]. Its activation requires both a priming signal, typically mediated by NF-κB, and a secondary trigger, such as mtROS, extracellular ATP, or microbial metabolites. Persistent exposure to biofilm-derived signals can sustain this dual activation, leading to continuous caspase-1–dependent maturation of IL-1β and maintenance of low-grade inflammatory signaling [39]. Collectively, PRR activation not only initiates immune responses but also reshapes epithelial metabolism, creating a feed-forward loop in which metabolic dysfunction reinforces inflammatory signaling.

### 4.3. Anti-Inflammatory Resilience and Limits of Epithelial Regulation

**Figure 4:**
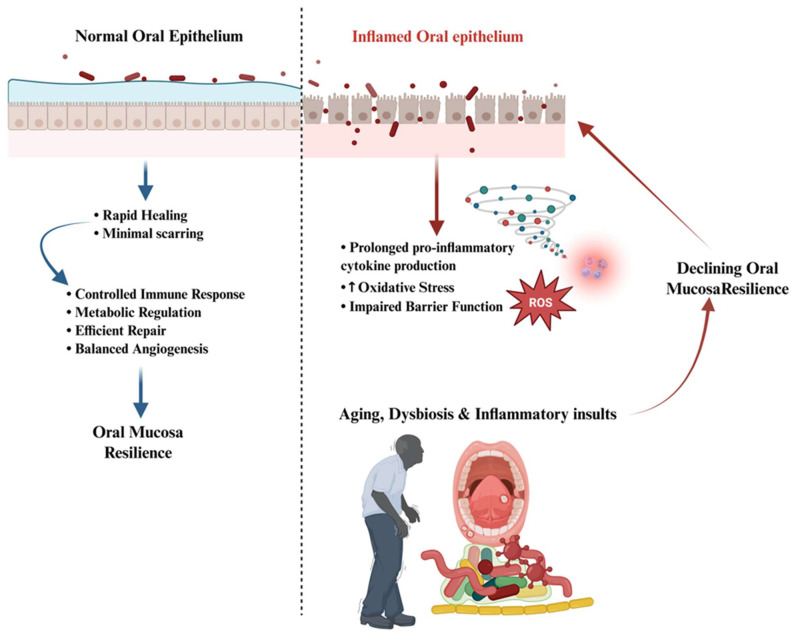
Oral mucosal resilience vs. inflammatory dysregulation. Physiological oral epithelium promotes controlled immune responses, balanced metabolism, and efficient repair, ensuring mucosal resilience. In contrast, inflamed epithelium exhibits impaired barrier function, oxidative stress, and sustained cytokine production, driven by aging, dysbiosis, and chronic inflammatory insults. Created in BioRender. Palumbo, P. (2026) https://BioRender.com/ks46uk1 (accessed on 7 May 2026).

A distinctive characteristic of the oral mucosa is its intrinsic resistance to chronic inflammation and fibrosis, despite constant exposure to microbial and mechanical stress. This resilience is supported by tightly regulated immunometabolic programs that limit excessive inflammatory activation while promoting rapid tissue repair (Figure 4). Key regulatory mechanisms include efficient antioxidant responses mediated by redox-balancing systems, as well as metabolic flexibility that enables the restoration of oxidative phosphorylation following transient inflammatory activation. In addition, cellular processes such as autophagy and mitophagy contribute to the clearance of damaged mitochondria, preventing excessive mtROS accumulation and limiting inflammasome activation. However, this regulatory capacity is not unlimited. Aging, sustained dysbiosis, and repeated inflammatory insults progressively erode epithelial metabolic flexibility and resolution mechanisms. As a consequence, epithelial cells exhibit prolonged activation of NF-κB and increased production of pro-inflammatory mediators, accompanied by alterations in barrier integrity [67]. A critical aspect of redox homeostasis is the coupling between glycolytic reprogramming and the pentose phosphate pathway (PPP). Increased glycolytic flux can redirect glucose-6-phosphate into the PPP, enhancing the production of Nicotinamide adenine dinucleotide phosphate (reduced form) (NADPH), which is essential for antioxidant defense systems, including glutathione and thioredoxin pathways [68,69,70]. While this mechanism initially supports redox balance, prolonged metabolic stress may impair PPP efficiency or increase NADPH demand beyond its capacity [71]. As a result, antioxidant defenses become insufficient, promoting oxidative stress, mitochondrial dysfunction, and sustained inflammatory signaling [60,72].

### 4.4. Epithelial Immunometabolic Dysfunction as a Source of Chronic Inflammation

Disruption of epithelial metabolic homeostasis transforms the oral barrier into a persistent source of inflammatory signals. Impaired mitochondrial function sustained glycolytic reprogramming, and defective regulation of redox balance collectively promote barrier dysfunction and increased permeability. Under these conditions, enhanced microbial translocation and continuous PRR engagement amplify NF-κB signaling and inflammasome activation [65,66]. The resulting secretion of cytokines, including IL-1β, IL-6, and IL-8, together with damage-associated molecular patterns (DAMPs), contributes to both local inflammation and systemic immune activation.

Importantly, this process can occur in the absence of overt tissue destruction. Instead, low-level but persistent epithelial immunometabolic activation provides a continuous source of inflammatory input, reinforcing chronic low-grade inflammation at the organismal level. Thus, epithelial immunometabolic dysfunction represents a key mechanism through which the oral barrier contributes to sustained systemic inflammatory tone.

## 5. Oral–Systemic Immunometabolic Crosstalk

Chronic inflammation is increasingly recognized as a multi-organ process sustained by continuous input from peripheral tissues, rather than being driven solely by systemic immune compartments. Barrier tissues exposed to persistent environmental and microbial stress act as active interfaces capable of generating and disseminating inflammatory signals. In this context, the oral mucosa emerges as a significant contributor to systemic inflammatory tone through mechanisms that integrate immune activation and metabolic regulation.

### 5.1. Microbial Translocation and Sustained Systemic Inflammation

Compromise of oral barrier integrity may facilitate the translocation of microbial components and metabolites across the epithelial interface into circulation. Even low-level repeated exposure to these signals may sustain chronic activation of innate immune pathways. Microbial ligands such as LPS engage PRRs, including TLRs on circulating immune cells, activating NF-κB-dependent signaling pathways and potentially promoting systemic cytokine production. In parallel, metabolic stress at the barrier level contributes to the release of mitochondrial DAMPs, including mitochondrial DNA and other pro-inflammatory signals, which further amplify innate immune activation [2,73].

Collectively, these processes establish a state of persistent, low-grade immune stimulation, linking localized barrier dysfunction to broader systemic inflammatory responses.

### 5.2. Salivary and Gingival Crevicular Mediators in Systemic Dissemination

Building on their local immunological functions at the oral barrier, saliva and gingival crevicular fluid also may serve as mediators of systemic inflammatory dissemination [15]. Beyond cytokines and chemokines, these biofluids contain microbial products, metabolites, and extracellular vesicles that can access circulation and influence immune responses at distant sites.

Extracellular vesicles derived from epithelial and immune cells carry bioactive cargo, including microRNAs, lipids, and mitochondrial components, which may modulate cellular responses systemically [74,75]. For instance, specific microRNAs involved in inflammatory regulation can influence nuclear factor kappa-light-chain-enhancer of activated B cells (NF-κB) signaling and metabolic pathways in recipient cells, thereby potentially shaping immune responses beyond the oral cavity [76]. In addition, mitochondrial DNA and other damage-associated molecules present in saliva and gingival crevicular fluid can enter the systemic circulation and activate innate immune pathways, including inflammasome signaling. Through these mechanisms, oral-derived mediators extend localized immunometabolic signals to distal tissues, contributing to organism-wide immune activation.

From a translational perspective, the identification of measurable biomarkers is essential to link local immunometabolic alterations to systemic outcomes. Several molecular and clinical biomarkers, including inflammatory cytokines (IL-1β, IL-6, TNF-α), mitochondrial stress indicators such as mtROS, and soluble mediators detectable in saliva and gingival crevicular fluid, may serve as indicators of oral immunometabolic dysfunction and its potential systemic impact [77,78,79].

### 5.3. Cross-Barrier Immune Networks and Systemic Integration

Inflammatory signaling originating from the oral barrier is integrated into broader cross-organ immune networks that connect multiple barrier sites and metabolic tissues [80,81]. Oral-derived microbial components and metabolites can influence gut microbiota composition, intestinal permeability, and downstream immune responses, contributing to interconnected axes such as the oral–gut axis [2].

In parallel, circulating inflammatory mediators and metabolic signals affect distal tissues, including the liver, vascular endothelium, and central nervous system. These interactions may link oral immunometabolic activity with processes such as metabolic dysregulation, endothelial activation, and neuroinflammation.

A key feature of this systemic integration is the progressive modulation of immune responsiveness driven by repeated low-level stimuli. Continuous exposure to microbial ligands and metabolic signals can promote a primed state in circulating immune cells, lowering activation thresholds and favoring sustained inflammatory responsiveness.

### 5.4. Barrier-Derived Signals in Systemic Inflammation

The cumulative effect of microbial translocation, dissemination of inflammatory mediators, and cross-organ signaling is the reinforcement of chronic low-grade systemic inflammation. Rather than triggering acute responses, repeated subthreshold stimuli may maintain continuous activation of key inflammatory pathways, including NF-κB and inflammasome signaling. Over time, this sustained activation may promote immune priming, altered metabolic regulation, and a reduced capacity for resolution of inflammation. In this framework, the oral mucosa functions as a persistent immunometabolic interface whose impact on systemic inflammation depends on the balance between adaptive regulation and chronic activation.

Collectively, oral–systemic immunometabolic crosstalk represents a mechanistic pathway through which localized barrier dysfunction shapes long-term inflammatory trajectories and contributes to the development of chronic inflammatory and metabolic diseases.

## 6. Barrier Immunometabolism and Chronic Inflammation

Chronic low-grade inflammation emerges from the sustained integration of immune activation, metabolic stress, and impaired resolution mechanisms.

Increasing evidence indicates that barrier tissues exposed to continuous environmental and microbial challenges act as persistent sources of inflammatory input, rather than passive targets of systemic immune dysregulation. Within this framework, the oral mucosa exemplifies how localized immunometabolic processes can drive organism-wide inflammatory responses [6].

### 6.1. Barrier Immunometabolic Drivers of Persistent Inflammatory Signaling

Barrier tissues rely on immunometabolic plasticity to maintain function under conditions of constant stimulation [2]. At the oral mucosa, epithelial and immune cells dynamically adjust their metabolic state in response to microbial ligands, mechanical stress, and inflammatory cues. This adaptation involves the coordinated activation of signaling pathways, including NF-κB, mTOR, and HIF-1α, which couple immune activation to metabolic reprogramming. Under physiological conditions, these responses are transient and support host defense and tissue repair. However, chronic stimulation drives sustained metabolic reprogramming characterized by increased glycolytic reliance, altered redox homeostasis, and progressive mitochondrial dysfunction. These changes promote the accumulation of mtROS and the release of DAMPs [39]. A key consequence of this metabolic stress is the activation of the NLRP3 inflammasome, linking mitochondrial dysfunction to persistent IL-1β production [65,66]. Continuous engagement of these pathways establishes a feed-forward loop in which metabolic dysregulation reinforces inflammatory signaling, favoring the maintenance of low-grade rather than resolving inflammation [82].

### 6.2. Oral Barrier Activation and Systemic Inflammatory Tone

Persistent immunometabolic activation at the oral barrier may contribute to systemic inflammatory tone through multiple mechanisms [23]. Repeated low-level microbial translocation provides continuous stimulation of circulating immune cells via PRRs, sustaining NF-κB-dependent cytokine production. In parallel, the dissemination of bioactive mediators, including cytokines, microbial metabolites, extracellular vesicles, and mitochondrial components, extends local signals to distal tissues. These mediators influence immune and metabolic responses in organs such as the liver, vasculature, and central nervous system.

Importantly, chronic exposure to subthreshold inflammatory stimuli promotes a primed state in innate immune cells, characterized by heightened responsiveness to secondary challenges. This process contributes to sustained inflammatory activity even in the absence of overt infection or tissue damage.

Collectively, these mechanisms illustrate how localized barrier activation can shape systemic immune homeostasis and drive chronic low-grade inflammation.

### 6.3. Implications for Chronic Inflammatory Diseases

The persistence of low-grade inflammation is a defining feature of aging and multiple chronic diseases, including metabolic syndrome, cardiovascular disorders, and neurodegeneration. Barrier-derived immunometabolic signaling provides a mechanistic link between continuous environmental exposure and long-term inflammatory burden.

Within this framework, key molecular nodes emerge as central regulators of inflammatory persistence, including HIF-1α–driven metabolic reprogramming, mtROS-dependent signaling, and NLRP3 inflammasome activation. Dysregulation of these pathways promotes the transition from adaptive immune responses to chronic, self-sustaining inflammation [39,65,66].

Understanding how these processes originate and evolve at barrier tissues may provide critical insight into early drivers of systemic inflammation and identify novel opportunities for therapeutic intervention. The oral mucosa, given its accessibility and high degree of immunometabolic activity, represents a valuable model for investigating these mechanisms.

### 6.4. Clinical and Epidemiological Evidence Supporting the Oral–Systemic Inflammatory Axis

Human studies provide important evidence supporting an association between oral inflammatory conditions and systemic low-grade inflammation, although the strength and directionality of these relationships remain incompletely defined. In particular, periodontal disease represents one of the most extensively studied clinical models linking local barrier inflammation to systemic immune activation [83].

Evidence indicates that individuals with periodontal inflammation exhibit elevated circulating inflammatory biomarkers [77,84,85]. These findings suggest that oral inflammatory burden may contribute to systemic inflammatory tone, potentially through the dissemination of inflammatory mediators or microbial components into the circulation. Notably, even moderate forms of periodontal disease have been associated with detectable increases in systemic inflammatory markers, supporting the concept of cumulative low-grade exposure [83].

Epidemiological studies further reinforce these observations, demonstrating associa-tions between oral inflammatory conditions and a range of systemic disorders, including cardiovascular disease, type 2 diabetes, metabolic syndrome, and neuroinflammatory conditions [86]. Although these associations are consistent and biologically plausible, they are influenced by confounding factors.

Longitudinal studies provide additional insights by suggesting that chronic oral inflammation may precede and contribute to systemic disease development. In particular, persistent periodontal inflammation has been associated with increased risk of cardio-vascular events, dysregulation of glucose metabolism, and progression of metabolic and inflammatory disorders over time [46,87,88,89].

Importantly, interventional studies offer some of the most compelling evidence supporting a functional link between oral and systemic inflammation. Periodontal treatment has been shown to reduce systemic levels of CRP, IL-6, and other inflammatory mediators, as well as to improve endothelial function and metabolic parameters in certain patient populations [90,91,92]. Although these effects are not universally consistent, they suggest that modulation of oral inflammation may influence systemic inflammatory burden.

Taken together, clinical and epidemiological evidence indicates that oral inflammatory conditions are associated with systemic low-grade inflammation and related disorders. However, while these findings support the biological plausibility of the oral–systemic axis, direct causal relationships and precise mechanistic pathways remain to be fully established.

## 7. Experimental Models for Studying Oral Immunometabolism

**Figure 5:**
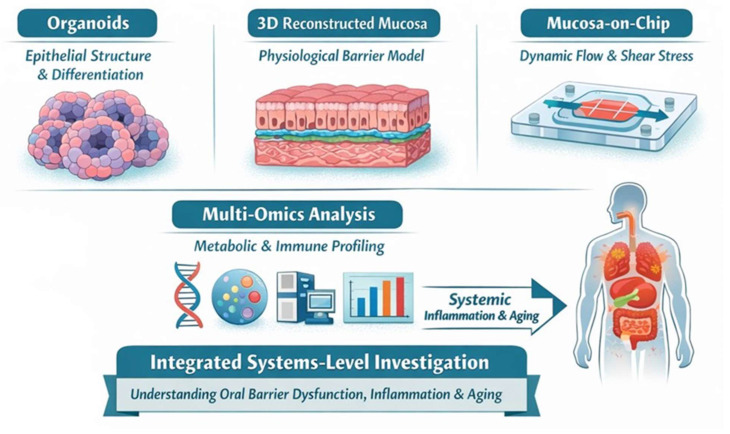
Experimental platforms for studying oral immunometabolism. Organoids provide insight into epithelial structure and cellular differentiation, while three-dimensional reconstructed mucosa models reproduce key features of the physiological epithelial barrier. Microfluidic mucosa-on-chip platforms enable the study of dynamic conditions such as flow and shear stress. Integrated multi-omics approaches support comprehensive analysis of metabolic and immune states.

Dissecting the mechanisms underlying barrier-derived immunometabolic signaling requires experimental systems capable of capturing the complex interplay between epithelial cells, immune populations, and microbial communities. Traditional in vitro models have provided foundational insights into individual signaling pathways but fail to recapitulate the spatial organization, metabolic gradients, and persistent stimulation that characterize barrier tissues in vivo. Recent advances in three-dimensional culture systems, microfluidic platforms, and integrative omics technologies offer new opportunities to investigate oral barrier immunometabolism as a driver of sustained inflammation [93,94,95]. Figure 5 provides an overview of these experimental platforms, highlighting how each model captures distinct aspects of oral barrier biology while contributing to an integrated mechanistic framework.

### 7.1. In Vitro Models of Oral Epithelial Immunometabolism

Two-dimensional epithelial culture systems have been instrumental in studying PRR signaling, NF-κB activation, and oxidative stress responses. However, these models mainly reflect acute conditions and do not capture the sustained activation characteristic of chronic low-grade inflammation.

Three-dimensional reconstructed mucosa and organoid systems provide improved physiological relevance by preserving epithelial stratification, barrier function, and aspects of tissue-specific metabolic programming [94,96,97,98]. These models enable the investigation of how prolonged microbial exposure reshapes epithelial metabolism and inflammatory responses over time.

### 7.2. Co-Culture Systems and Microbial–Immune Interactions

Modeling chronic inflammation at barrier tissues requires experimental systems capable of integrating epithelial, immune, and microbial components [93,98]. Advanced co-culture models enable controlled interactions between oral biofilms, epithelial layers, and innate immune cells, including neutrophils, macrophages, and dendritic cells. Compared with monoculture systems, these platforms more effectively reproduce the dynamic crosstalk that drives immunometabolic responses at the oral barrier.

A key advantage of co-culture systems lies in their ability to support sustained rather than acute stimulation. Continuous or repeated exposure to microbial communities enables the investigation of how persistent signaling reshapes epithelial and immune cell metabolism and promotes prolonged cytokine production while altering barrier integrity. Consequently, these systems are particularly suited to studying immune priming, and failure of resolution.

However, current co-culture models remain constrained by limited control over long-term stability and system complexity. However, these models are limited by challenges in maintaining stable, physiologically relevant interactions among multiple cell types over time and by the lack of full integration of spatial and systemic components.

Despite these limitations, co-culture platforms represent a critical intermediate be-tween reductionist in vitro systems and complex in vivo models, providing essential in-sight into how sustained microbial–immune interactions contribute to barrier-associated immunometabolic dysfunction.

To more faithfully reproduce the dynamic and sustained conditions that characterize barrier-associated inflammation, these approaches have been further advanced through microfluidic and barrier-on-chip technologies that integrate spatial organization with controlled environmental parameters.

### 7.3. Microfluidic Platforms and Barrier-on-Chip Technologies

Microfluidic barrier-on-chip systems incorporate dynamic parameters essential for modeling chronic inflammation, including fluid flow, shear stress, nutrient gradients, and repeated microbial exposure. Oral mucosa-on-chip devices enable real-time monitoring of epithelial permeability, inflammatory mediator release, immune cell recruitment, and metabolic flux under conditions that closely mimic physiological stress [95,99,100].

These platforms provide unique opportunities to investigate how cyclical mechanical and microbial stimuli interact with immunometabolic pathways to sustain low-grade inflammation. Integration with biosensors and live imaging enables high-resolution monitoring of inflammatory dynamics, distinguishing transient from persistent responses.

While these systems effectively capture dynamic physical and cellular interactions, deeper mechanistic insight requires complementary molecular profiling approaches.

### 7.4. Multi-Omics Approaches and Spatial Analysis

High-resolution analytical techniques, including spatial transcriptomics, single-cell sequencing, and metabolomics, have transformed the study of barrier-associated inflammation [101,102,103]. When applied to oral tissues and advanced in vitro systems, these methodologies reveal how immunometabolic programs are spatially organized and how specific cell populations contribute to inflammatory signaling. Integration of multi-omics datasets enable identification of cell-specific metabolic reprogramming, inflammatory signaling, and immune–epithelial interactions. Collectively, these strategies provide critical insight into how cellular-level processes scale to organism-wide inflammatory trajectories, enabling a systems-level understanding of barrier-derived signaling.

### 7.5. Integrative Modeling of Chronic Barrier-Derived Inflammation

No model fully recapitulates the complexity of chronic inflammation at barrier tissues. However, the integration of advanced culture models, microfluidic platforms, and omics-based approaches offers a comprehensive framework for investigating how immunometabolic stress accumulates and persists over time. Such integrative strategies provide a powerful approach for elucidating the mechanisms linking barrier dysfunction to systemic inflammation and for identifying novel therapeutic targets.

To facilitate a comparative evaluation of the experimental systems discussed in this section, Table 1 summarizes their biological complexity, time scale, key output measures, limitations, and suitability for modeling chronic low-grade inflammation.

## 8. Conclusions

Chronic low-grade inflammation arises from sustained interactions between immune activation, metabolic stress, and impaired resolution mechanisms. Barrier tissues exposed to continuous environmental challenges play a critical role in this process by acting as persistent sources of inflammatory signals.

In this review, we highlight how the oral mucosa functions as an immunometabolic interface in which epithelial cells, immune populations, and microbial communities converge to sustain low-grade inflammatory signaling. Key molecular mechanisms, including HIF-1α–driven metabolic reprogramming, mitochondrial dysfunction associated with mtROS production, and activation of the NLRP3 inflammasome, emerge as central regulators of this process [39].

Oral–systemic crosstalk further amplifies these effects through microbial translocation, dissemination of inflammatory mediators, and integration within cross-organ immune networks. Together, these interactions support a model in which sustained immunometabolic engagement at the oral barrier contributes to systemic inflammatory tone independently of overt pathology.

This framework extends rather than replaces existing models such as the oral–systemic axis by shifting the focus from microbial dissemination alone to the intrinsic immunometabolic activity of the oral barrier. In this context, the oral mucosa emerges as an active regulator of systemic inflammatory tone through locally sustained metabolic and immune integration. Furthermore, the link between local oral immunometabolic activity and systemic inflammation is likely governed by cumulative exposure and threshold effects. Repeated low-level stimulation at the oral barrier may progressively prime immune responses. Once a critical threshold is reached, these signals may drive sustained systemic inflammation.

Notably, a substantial portion of the mechanistic evidence discussed in this review is derived from intestinal or systemic models, whereas direct experimental validation in the oral mucosal environment remains relatively limited. This highlights an important gap in current knowledge and underscores the need for dedicated oral-specific investigations.

Accordingly, the proposed mechanistic framework should be interpreted as partly extrapolated from other barrier systems and requires further validation in oral-specific contexts. Future studies integrating advanced experimental approaches, including co-culture systems, organoids, and microfluidic platforms, will be essential to investigate oral immunometabolism under conditions of chronic low-grade inflammation and to validate these mechanisms in a physiologically relevant oral environment.

Within the multifactorial framework of inflammaging, the oral mucosa should be considered as one of several contributing barrier sites, interacting with systemic metabolic and immune processes rather than acting as an isolated or predominant driver.

Despite growing evidence supporting a link between oral immunometabolic activity and systemic inflammation, this relationship remains incompletely defined. Current knowledge is largely derived from mechanistic insights obtained in experimental and preclinical models, together with associative findings from clinical studies. In addition, the complexity of host–microbiota interactions and their systemic integration remain only partially understood. Therefore, the proposed framework should be considered an emerging and testable model rather than a fully established causal paradigm. Further studies are needed to address unresolved questions and to define the quantitative contribution of oral barrier immunometabolism within the broader context of systemic inflammatory regulation. Importantly, the mechanisms described reveal potential targets for therapeutic intervention. Modulation of inflammasome activity, restoration of mitochondrial function, and regulation of microbiota-derived metabolites represent promising strategies for limiting chronic inflammation at its peripheral origins.

**Figure 6:**
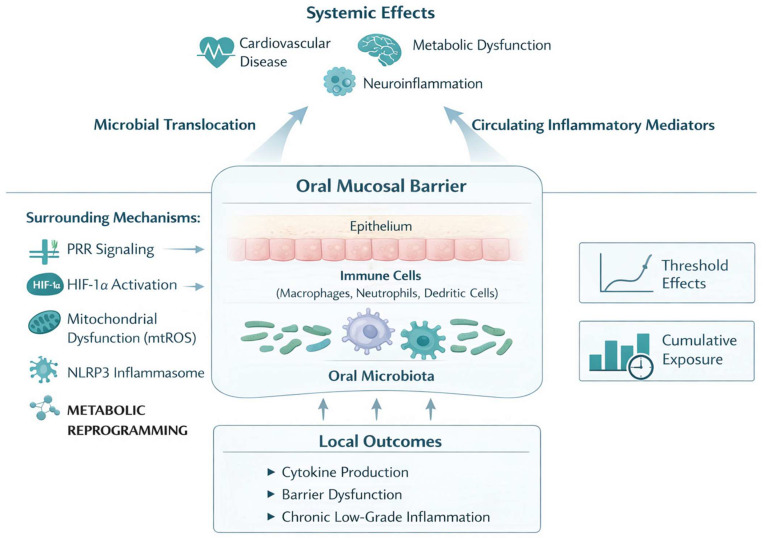
Integrated schematic model of oral mucosal immunometabolic dysfunction and its systemic implications. The oral mucosal barrier is depicted as a central immunometabolic interface composed of epithelial cells, resident immune populations (including macrophages, neutrophils, and dendritic cells), and the oral microbiota. Local barrier dynamics are influenced by interconnected molecular pathways, including pattern recognition receptor (PRR) signaling, HIF-1α activation, mitochondrial dysfunction with mtROS production, NLRP3 inflammasome activation, and metabolic reprogramming. Sustained activation of these pathways contributes to key local outcomes, including cytokine production, barrier dysfunction, and chronic low-grade inflammation. These processes may facilitate microbial translocation and the release of circulating inflammatory mediators, thereby linking local oral alterations to systemic inflammatory processes. Systemic effects include cardiovascular disease, metabolic dysfunction, and neuroinflammation. The model also incorporates key conceptual elements discussed in this review, including cumulative exposure and threshold effects, highlighting how repeated low-level stimulation may progressively drive the transition toward chronic low-grade inflammation over time.

Taken together, the oral mucosa provides a valuable and accessible model for understanding how barrier-associated immunometabolic processes shape long-term inflammatory trajectories. Targeting these pathways may offer new opportunities to mitigate inflammaging and its associated diseases, positioning barrier immunometabolism as a central determinant of chronic inflammation. A comprehensive schematic overview of the proposed framework is presented in Figure 6, illustrating the integration of local immunometabolic processes at the oral barrier with downstream local and systemic outcomes, including the roles of cumulative exposure and threshold effects.

## Figures and Tables

**Table 1:** Comparison of experimental models used to investigate oral immunometabolism, highlighting their relative complexity, time scale, key output measures, limitations, and suitability for modeling chronic low-grade inflammation.

Model	Biological Complexity	Time Scale	Suitable for Chronic Low-Grade Inflammation	Key Outputs	Limitations
2D epithelial cultures	Low	Short-term	Low	Cytokine production, signalling pathways	Lack of tissue architecture, acute responses only
Co-culture systems	Moderate/High	Short/Medium- term	Moderate/High	Immune–epithelial interactions, cytokines	Simplified microenvironment, limited long-term stability
3D organoids	Medium/High	Medium-term	Moderate	Barrier function, differentiation, metabolic activity	Limited immune and vascular components
Microfluidic (organ-on-chip)	High	Long-term	Very high	Dynamic responses, permeability, metabolic flux, immune interactions	Technical complexity
In vivo models	Very high	Long-term	High	Systemic responses, disease progression, chronic inflammation	Limited mechanistic resolution and experimental control

## Data Availability

No new data were created or analyzed in this study. Data sharing is not applicable to this article.

## Abbreviations

The following abbreviations are used in this manuscript:

Ab	Antibody
APC	Antigen presenting cell
ATP	Adenosine triphosphate
DAMPs	Damage-associated molecular patterns
FDC	Follicular dendritic cells
FMO3	Flavin-containing monooxygenase 3
GLUT1	Glucose Transporter Type 1
GPR43	G protein–coupled receptors
HEV	High endothelial venules
HIF-1α	Hypoxia-inducible factor-1α
IL	Interleukin
LPS	Lipopolysaccharide
MAPK	Mitogen-activated protein kinase
mTOR	Mechanistic target of rapamycin
mtROS	Mitochondrial reactive oxygen species
NAD+	Nicotinamide adenine dinucleotide
NADH	Nicotinamide adenine dinucleotide hydride
NADPH	Nicotinamide adenine dinucleotide phosphate (reduced form)
NF-kB	Nuclear factor kappa-light-chain-enhancer of activated B cells
NLRP3	NLR family pyrin domain containing 3
NLRs	Nucleotide-binding oligomerization domain (NOD)-like receptors
PPP	Pentose phosphate pathway
PRRs	Pattern-recognition receptors
ROS	Reactive oxygen species
SCFAs	Short-chain fatty acids
sIgA	Secretory Immunoglobulin A
TLRs	Toll-like receptors
TMAO	Trimethylamine N-oxide
TNF	Tumor necrosis factor
